# Advancing sex and gender competency in medicine: sex & gender women’s health collaborative

**DOI:** 10.1186/2042-6410-4-11

**Published:** 2013-06-01

**Authors:** Alyson J McGregor, Kimberly Templeton, Mary Rojek Kleinman, Marjorie R Jenkins

**Affiliations:** 1 Emergency Medicine, Warren Alpert Medical School of Brown University, Rhode Island Hospital, Providence, RI, USA; 2 Orthopedic Surgery, University of Kansas Medical Center, Kansas City, KS, USA; 3 Graduate Fellow in Sociology, Loyola University Chicago, Chicago, IL, USA; 4 Women in Health and Science, and Chief Scientific Officer, Laura W. Bush Institute for Women’s Health at the Texas Tech University Health Sciences Center, Amarillo, TX, USA; 5 Sex and Gender Women’s Health Collaborative (SGWHC) Working Group, USA

**Keywords:** Medical education, Sex and gender differences, Women’s health

## Abstract

Research conducted to date has deepened our understanding of sex and gender differences in the etiology, diagnosis, treatment, and outcomes for many conditions that affect both women and men. The Sex and Gender Women’s Health Collaborative (SGWHC) is supported by the coordinated efforts of our founding partners: the American Medical Women’s Association, the American College of Women’s Health Physicians and Society for Women’s Health Research to address the gaps in medical education with regard to sex and gender competency in the care of women. The SGWHC initiated and continues to build a novel digital resource library of sex and gender specific materials to be adopted and adapted into medical education and clinical practice, residing @ http://www.sgwhc.org. This article presents a case for the inclusion of sex and gender focused content into medical curricula and describes a means for students, faculty, and practitioners to access a centralized, interactive repository for these resources.

## Introduction

The emerging discipline of sex and gender-specific medicine evolved out of the field of women’s health, and addresses health care issues beyond hormones and reproduction. Sex and gender-specific medicine is based on the science of normal human functioning and how experiences of the same disease are similar and differ as a function of gender and biological sex [1,2]. This emerging discipline goes beyond women’s health in that it also informs the teaching of men’s health. For example, men, in general, have shorter life spans, present decades earlier with coronary artery disease, and are more likely to have their osteoporosis underdiagnosed and untreated [3-5]. The ability to customize patient-specific strategies based on individual genes and proteins so as to better predict, prevent, diagnose, and treat subtypes of disease rather than using the “one-size-fits-all” approach is improving due to the advances in biotechnology [6]. The emerging concepts of Individualized Medicine offer tools to incorporate the knowledge of patient sex and gender to search for answers to common health problems. In other words, integrating sex and gender into medical education and clinical practice will provide more personalized, evidenced-based care for all. For this to occur, sex and gender competency must be a goal of medical education. The Sex and Gender Women’s Health Collaborative was founded to assist faculty and students to develop this competency by establishing a digital repository of teaching tools, curricular materials, and other resources in an open access online format.

## Review

### Historical perspective

**Figure 1:**
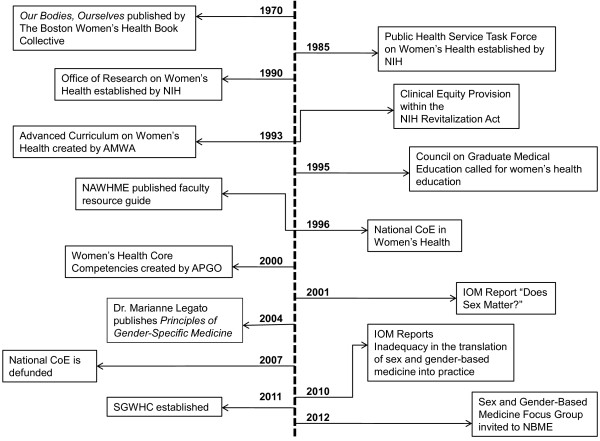
**Key historical contributions to the evolution of sex and gender in medicine.** Source: Adapted from developing timeline within the Sex and Gender Women’s Health Collaborative.

To understand the significance of the evolution of sex and gender within a biomedical construct, it is important to recognize the historical transition in the concept of women’s health (Figure 1). The lay women’s health movements of the 1960s and 1970s drew attention to many women’s health needs that had not previously been addressed, especially those related to reproductive health issues [7]. One of the most influential and enduring groups was The Boston Women’s Health Book Collective. They published a pamphlet in 1970 to educate women about their bodies, which became the groundbreaking book, *Our Bodies, Ourselves,* and which has now been translated and adapted into 25 languages [8]. This was followed in the 1980s by the entry of larger numbers of women into the medical profession. The combination of these factors led to a broader awareness both within and outside of the medical profession of some of the biases and inequities related to women’s health. For example, despite the fact that women make up over 50% of the population and are the major consumers of health care and prescription drugs, medical research has historically focused on men [9]. When most health-related knowledge is a result of studies only including men, this bias is translated into medical education and ultimately into clinical practice.

This gender bias has roots in how medical knowledge was organized and produced going back at least into the nineteenth century [10]. Prior to World War II, women were categorized as “protected subjects” in clinical investigations, particularly drug trials, out of fear of unforeseen teratogenic harm to the fetus among pregnant women. Women’s fluctuating hormonal status raised uncertainty about any comparisons between subjects, but the variability in men’s hormone levels was ignored; there was also concern that controlling for women’s hormonal differences would require increased sample sizes and study expense [11]. Despite the recognition of physiologic, anatomic, and metabolic differences between the sexes, there was an implicit assumption that outcomes in men would be adequate proxies for outcomes in women.

In 1985, the National Institutes of Health (NIH) established a Public Health Service Task Force on Women’s Health that issued recommendations to increase attention to women’s health issues, leading to guidelines for inclusion of women in NIH-funded extramural research [12]. The NIH established the Office of Research on Women’s Health (ORWH) in 1990 to assure that women’s health issues were adequately addressed, and appropriately represented in federally-supported research. Offices of women’s health were also established at the Department of Health and Human Services (DHHS), Centers for Disease Control (CDC), and United States Food and Drug Administration (FDA). The 1993 NIH Revitalization Act included a Clinical Equity Provision to ensure that the efficacy of treatments for women would not be extrapolated from data derived from male participants but instead would be determined directly from research on women [13].

Beginning in the 1990s, a number of medical professional organizations began to develop women’s health curricula to address the gender gap in medical education beyond reproductive health issues. The American Medical Women’s Association (AMWA) created the Advanced Curriculum on Women’s Health in 1993. The Association of Professors of Gynecology and Obstetrics (APGO) initially developed Women’s Health Care Competencies for Medical Students in 2000, and later expanded this into a comprehensive set of competencies. The National Academy on Women’s Health Medical Education (NAWHME) published a resource guide for faculty in 1994 to assist faculty in developing women’s health curricula [14]. In 1995, the Council on Graduate Medical Education called for physicians to be educated about women’s health [15]. Other medical societies supported the publication of books, and facilitated the dissemination of educational materials. Within medical schools, residency programs and fellowships in women’s health were established, as were women’s health tracks, and individual courses (most often clinical clerkships) for undergraduate medical education [16]. At the federal level, National Centers of Excellence in Women’s Health were established beginning in 1996 at academic medical centers and community health organizations, with one of their goals being to address the gender gap in medical education.

Collectively, the newfound attention to women’s health expanded the knowledge base. The initial focus was on the documentation of differences between men and women, as well as attention to issues unique in women [14]. As differences in women began to be recognized - such as differences in the presentation of a myocardial infarction, an understanding started to emerge that social factors – especially gender - were increasingly important in the emergence, presentation, treatment, and outcomes of many illnesses. If social gender factors influenced women’s health, then they must also influence men’s health. Thus, as a more sophisticated understanding of women’s health developed, and as scientific evidence accumulated with research that more frequently included women, the discipline of sex and gender-specific medicine started to emerge [17]. Despite decades of progress, an Institute of Medicine (IOM) report in 2010 indicated an inadequacy in the translation of sex and gender-based science into practice [18].

### Sex and gender-specific medicine

“Sex” refers to biological differences between women and men, including chromosomes, sex organs, and hormonal profiles. “Gender” refers to socially constructed and enacted roles and behaviors which occur in a historical and cultural context and vary across societies and over time. *Every cell has a sex*. All individuals act in many ways that fulfill the gender expectations of their society. With continuous interaction between sex and gender, health is determined by both biology and the expression of gender.

The Institute of Medicine (IOM) reports, “Sex, that is being male or female, is an important basic human variable that should be considered when designing and analyzing studies in all areas and at all levels of biomedical and health related research” (IOM 2001, p.3). Whether a cell contains an XX or XY chromosome may have an impact on everything from regulation of gene expression in a cell line to efficacy or toxicity of pharmaceuticals in humans [19]. It is now evident that gender also has a significant role in disease and response to treatment. Therefore, the significance of sex, gender, and their interaction should be considered in the daily practice of patient care.

Important examples of sex and gender differences and their interaction include:

•Aspirin has different preventive effects in men and women. It prevents stroke but not myocardial infarction (MI) in women, while preventing MI but not stroke in men [20].

•Musculoskeletal disease has differing incidence and manifestations between sexes. For example, women have a higher incidence of osteoarthritis, osteoporosis, and non-contact sports injuries, such as anterior cruciate ligament tears [21].

•For the same number of cigarettes smoked, women are more likely than men to develop chronic obstructive pulmonary disease [22]. It is theorized that for a given increase in the thickness of airway mucosa, women suffer a greater degree of airflow limitation given their typically smaller stature and smaller airway caliber [22,23].

•Gender differences in morbidity and self-reported health status by women are thought to contribute to the increased use of medical services and higher outpatient expenditures compared to men, even when controlling for health status and other variables [24].

•Men and women experience stress differently based on gender roles, which has an impact on their neurobiology [25]. For example, in men, there is a significant positive correlation in perceived stress and physiological responses at work. However, in women, physiological stress levels at work seem to spill over into non-work situations. This interaction between stress from paid employment and unpaid work at home is important to consider in the study of women’s stress [26].

•For example, cortisol levels generally decrease in the evening among men, but not for women [27].

•Female mortality rates increased in 42.8 percent of counties, while male mortality rates increased in only 3.4 percent [28].

As sex and gender factors are considered in research and results are published, the transfer of scientific findings to practice is only possible if they are incorporated into medical education and training. Sex and gender can be integrated into medical education in many different ways - from student electives, to a longitudinal sex and gender “thread” that incorporates sex and gender into every area of student learning. Since sex and gender are two human variables that everyone possesses without exception, the longitudinal approach, while more ambitious, is optimal. In order to design sex and gender sensitive medical curricula, it is imperative that specific content areas are identified and educational resources are readily available to both educators and students. This makes a single, accessible digital site for such materials as provided within the Sex and Gender Women’s Health Collaborative (http://www.sgwhc.org) essential.

### The sex and gender women’s health collaborative

The Sex and Gender Women’s Health Collaborative (SGWHC) is a coordinated effort of the American Medical Women’s Association, the American College of Women’s Health Physicians, and Society for Women’s Health Research to address the gaps in medical education surrounding sex and gender specific care of women. While initially focused on women’s health, a sex and gender approach is also expected to lead to a critical assessment of our understanding of men’s health. The mission of the SGWHC is to foster sex and gender competency in all medical and health education institutions and across medical care. To achieve this, the SGWHC is currently working towards:

1) Assembling and organizing sex and gender based content aimed at improving the clinical care of women;

2) Creating a comprehensive, digitally accessible repository of evidence-based sex and gender specific resources for students and faculty in the medical and health care professions;

3) Promoting an understanding of the impact that sex and gender have on individual health status, clinical outcomes, and healthcare systems; and

4) Facilitating inclusion of sex and gender sensitivity into medical curricula and the training of future physicians.

The digital resource library -- http://www.sgwhc.org – is in the early stages of collecting relevant sex and gender material in the form of journal articles, abstracts, PowerPoint presentations, book reviews, reports, guidelines, case studies and other teaching tools. The site also lists other resources such as books, textbooks, conferences featuring sex and gender content, continuing medical education activities, and a blog for news and discussion. Future plans include the establishment of a peer review process, journal clubs, and scientific seminars. The site will have the opportunity to enable faculty to locate sex and gender specific materials for integration into their course materials and will enable students to be proactive about the inclusion of sex and gender content into their medical education. The materials are useful to students, educators, fellows, researchers, and clinical practitioners in other fields such as nursing, pharmacy, and physical therapy.

This project began primarily as a collaboration of national leaders in women’s health and has expanded to include international expertise. Collaborating organizations, such as medical schools, professional organizations, National Centers of Excellence in Women’s Health, women’s health institutes, societies, and government organizations, are listed on the site.

Prior to the launch of this project, there was no central, organized, easily accessible place to find the sex and gender specific information that has been researched, published, and distributed over the last 20 years. Other web-based research and educational resources have since emerged and include Stanford University’s Gendered Innovations (available at http://genderedinnovations.stanford.edu) whose goal is to employ sex and gender analysis as a resource for knowledge and technology [29,30] as well as Canadian Institute of Gender Health: What a Difference Sex and Gender Make (available at http://www.cihr-irsc.gc.ca/e/44082.html.) a peer-reviewed casebook that illustrates the gender and sex contributions to health research [29,31]. The SGWHC aims to provide the tools and resources to improve medical education by enhancing the discussion of the impact of the most basic patient characteristic: being male or female.

### Licensing examinations

Another project of the SGWHC is to identify content areas for possible curriculum development, testing, and evaluation, while providing resources for these on the Collaborative website. Early in 2012, a group of thirty national thought leaders in women’s health and sex and gender-specific medicine, coordinated by the SGWHC, identified over 200 clinical areas for which there is an evidence base for sex and/or gender differences in etiology, incidence, presentation, outcome, or prevention. These topics, along with the listed references supporting sex and/or gender differences, will be published on the Collaborative website.

National licensing examinations, such as the United States Medical Licensing Exam® (USMLE®), can indicate the recommended scope of knowledge for all medical students. Identifying this content is especially significant for sex and gender-based topics, wherein material is covered in a variety of modules and clerkships, rather than in a defined course, as it spans all organ systems. In the late 2012, sample USMLE examination forms were reviewed to identify where this content is already covered and any missed opportunities that might be addressed, including differences in health and health maintenance, mechanism of disease, diagnosis, and management [32]. The group is now in the process of developing a report to the National Board of Medical Examiners. Similar initiatives concerning standardized examinations have occurred with other content areas. Efficacy of increased emphasis on and exposure to a specific area has been demonstrated in these other content areas [33]. However, in addition to enhancing the knowledge base in a given area, there needs to be continued clinical exposure during the course of training, as standardized examinations do not assess higher levels of medical decision-making or the use of knowledge in practice [34]. The sex and gender focused resources available to faculty by the SGWHC should facilitate the incorporation of this material both into the didactic as well as clinical teaching areas of the medical school experience.

## Conclusions

Over the past several decades, a significant international body of research on sex and gender differences in health and illness has developed and continues to grow. However, there has been too little information applied to clinical practice. Medical educators must aim to train medical students for sex and gender competency. The mission of SGWHC is to foster the integration of sex and gender content into medical education and training. As a start, individual faculty can update course materials to adequately represent this sex and gender evidence base. The content available from SGWHC.org will enable educators to download educational materials for direct use, adapt their existing materials, and contribute material for other health educators.

The future of clinical practice is oriented toward individualized patient care. Just as the development of biotechnologies allow us to provide individualized care based on a patient’s genetic or hormonal profiles, knowledge of sex and gender differences will allow us to provide care based on a patient’s social and psychological circumstances, because these factors also affect their health in profound ways.

## Competing interest

All authors listed have contributed sufficiently to the conceptualization and actualization of this project. All those who are qualified to be authors are listed in the author byline. There is, to the best our knowledge, no conflict of interest, financial or otherwise. We have included conflicts of interest, authors’ contributions and acknowledgements after our summary. All authors declare that they have no competing interests.

## Authors’ contributions

Author AJM conceived and outlined the review. Authors AJM, KT, MRK, and MRJ equally wrote major sections of the first draft of the manuscript. AJM performed the literature review. AJM and MRK made major changes to create the final draft of the manuscript. AJM prepared the Figure. All authors contributed to and have approved the final manuscript.

## Authors’ information

AJM, assistant professor of Emergency Medicine and Director of Women’s Health in Emergency Care Division at the Alpert Medical School of Brown University, Providence, RI; MRK, Graduate Fellow at the Center for Urban Research and Learning, Loyola University Chicago. MRJ, professor and associate dean for Women in Health and Science, and Chief Scientific Officer, Laura W. Bush Institute for Women’s Health at the Texas Tech University Health Sciences Center in Amarillo, TX; KT, professor of orthopedic surgery at University of Kansas Medical Center, Kansas City, KS.

AJM, MRK, MRJ, KT are members of the Sex and Gender Women’s Health Collaborative Working Group. J Werbinski, *Chair,*Medical Director of Borgess Women’s Health and assistant clinical professor of Obstetrics & Gynecology at Western Michigan University School of Medicine, Kalamazoo, MI; Jodi Godfrey, MS, RD, *SGWHC Program Coordinator and Web Editor.*
